# Transport pathways across the West African Monsoon as revealed by Lagrangian Coherent Structures

**DOI:** 10.1038/s41598-020-69159-9

**Published:** 2020-07-27

**Authors:** Coumba Niang, Ana Maria Mancho, Víctor José García-Garrido, Elsa Mohino, Belén Rodriguez-Fonseca, Jezabel Curbelo

**Affiliations:** 1 Instituto de Ciencias Matemáticas, Consejo Superior de Investigaciones Científicas (CSIC), C/ Nicolás Cabrera 15, Campus de Cantoblanco, 28049 Madrid, Spain; 2 Laboratoire de Physique de l’Atmosphére et de l’Océan Simón Fongang (LPAO-SF), Ecole Supérieure Polytechnique (ESP), Université Cheikh Anta Diop, BP 5085, Dakar-Fann, Senegal; 3 Departamento de Física y Matemáticas, Universidad de Alcalá, 28871 Alcalá de Henares, Spain; 4 Departamento de Fisica de la Tierra y Astrofisica, Universidad Complutense de Madrid (UCM), Madrid, Spain; 5 Departament de Matemàtiques, Universitat Politècnica de Catalunya (UPC), 08028 Barcelona, Spain

**Keywords:** Applied mathematics, Atmospheric dynamics

## Abstract

The West African Monsoon (WAM) system is the main source of rainfall in the agriculturally based region of the Sahel. Understanding transport across the WAM is of crucial importance due to the strong impact of humidity and dust pathways on local cloud formation. However, the description of this transport is challenging due to its 3D complex nature. Lagrangian Coherent Structures (LCS) simplify transport description across the WAM by providing a geometrical partition of the troposphere into domains. Air parcels within each domain have similar dynamical characteristics. LCS make it possible to achieve an integrated vision of transport pathways across this system. Using this approach we unveil new connections in the WAM system. In particular, we identify transport pathways between the Tropical Easterly Jet (TEJ) and the African Easterly Jet (AEJ). Furthermore, the clockwise circulation associated with the divergent upper part of the Sahara heat low is clearly delimitated. Additionally, we show the presence of mixing regions in the AEJ and the lower part of the TEJ that are linked to pathways to sources of dust and humidity.

## Introduction

The West African climate has been recognized as one of the hotspots in the Earth’s climate system^1, 2^. It is dominated by the West African Monsoon (WAM) system, one of the most complex monsoon systems on Earth in which land, ocean and atmosphere are highly coupled. The WAM is crucial for the population of the region. Its variability has a substantial impact on agriculture, livestock farming, water and food resources all of which strongly depend on rainfall, especially in the Sahel. Over this region, accumulated daily rainfall amounts vary from 2 to 16 mm per day during the rainy season^3, 4^. However, rainfall over the region shows variability at a wide range of time scales: from the decadal droughts during the 1970s and 1980s—which led to widespread famine—to intraseasonal fluctuations^5–8^. A comprehensive investigation of the WAM features is of prime importance for understanding and predicting the variability at those timescales as well as for the development of the fragile West African economy^7^.

**Figure 1:**
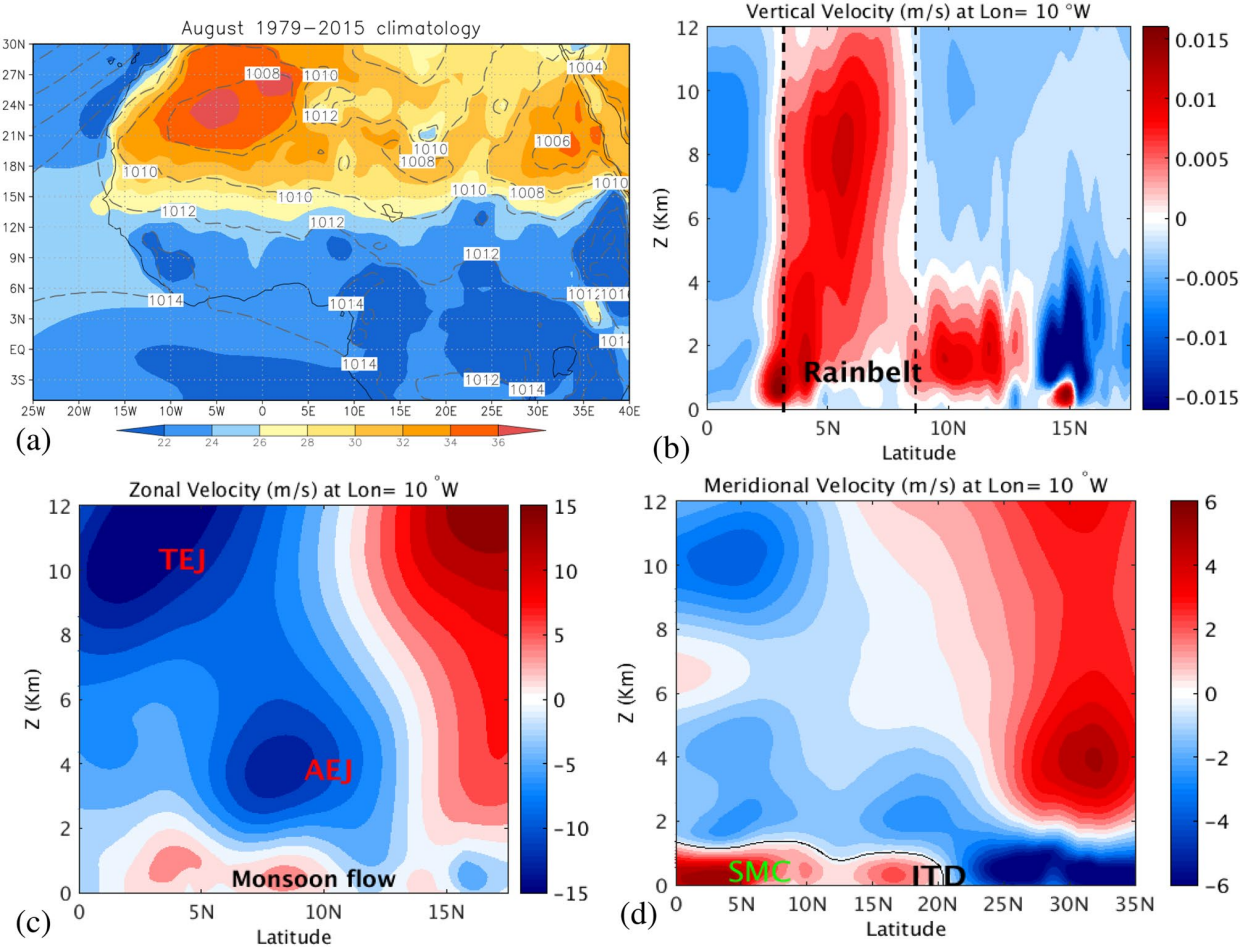
(**a**) Climatology of 2 m temperature (shaded, in °C) and mean sea level pressure (contour, in hPa) of ERA-Interim averaged for the month of August in the 1979–2015 period. Velocities at 10° W in m/s. (**b**) Vertical velocity component: positive (negative) values are updraft (downdraft) winds; (**c**) Zonal velocities: positive (negative) values are westerly (easterly) wind; (**d**) Meridional velocities: positive (negative) values indicate southerly (northerly) wind. All these data are averaged for the month of August over the 1979–2015 period. The rainbelt band located between 5° N and 15°N is represented in plot (**b**). In addition, the approximative location of the Tropical Easterly Jet (TEJ), the African Easterly Jet (AEJ) and the monsoon flow are labeled in plot (**c**) while the southerly Shallow Meridional Cell (SMC) and the InterTropical Discontinuity (ITD) are shown in plot (**d**). Map in panel (**a**) is done with Grid Analysis and Display System (GrADS) Version 2.2.0. The URL is http://cola.gmu.edu/grads/.

From an energetic point of view, monsoons are a manifestation of the ITCZ migration over tropical land regions. ITCZ is a facet of the direct overturning circulation exporting energy away from the tropics. The seasonal variation of the solar insolation drives the migration of the ITCZ towards the warmer hemisphere, so that the surplus of integrated moist static energy is exported across the Equator^9^. This simple picture is complicated in the case of monsoons by the presence of land masses, the different heat capacity of which makes the seasonal migration of the monsoon lag behind the sun’s position and also induce zonal asymmetries^10^. In the case of West Africa, further factors need to be considered: the development of the Atlantic cold tongue close to the Equator during boreal spring and summer, or the presence of the Saharan Heat low (see Fig. 1a) that limits the northward extension of the monsoon^4, 11, 12^. These factors play a role in shaping the different flows involved in the WAM, which, in turn, are key for transporting moisture and energy across the monsoon. From an Eulerian perspective, the average behaviour of such flows can be estimated as the climatology by computing the means of averages of daily flows over long periods. Fig. 1 shows such averaged flows for the month of August in the 1975–2015 time series. This month is considered as the peak of the monsoon over the Sahelian region^4^. The southerly part of the monsoon flow is visible below 1.5 km up to the Guinean coast at 5° N, where it converges to form a southerly Shallow Meridional Cell (Fig. 1d)^13^, which could be related to a shallow breeze circulation cell and to frictional deceleration of the flow as it reaches the coast^4, 14^.

The rainfall belt develops from 5° N and 15° N, with strong upward motions involving deep convection through the whole tropospheric column (Fig. 1b). Driven by geostrophic adjustment, the African Easterly Jet (AEJ) appears at mid-tropospheric levels (600 hPa, 4 km approximately) (Fig. 1c) and the Tropical Easterly Jet (TEJ) develops at the top of the troposphere (between 250 and 150 hPa, 11 km approximately)^15–17^. The AEJ is maintained by two diabatically forced circulations: dry convection to the north and moist convection to the south. In the mid-troposphere, the anticyclonic circulation associated with the diverging flow at the top of the Sahara heat Low (SHL) contributes to the maintenance of the African Easterly Jet (AEJ) and modulates its intensity^18^.

The ITCZ is a convergence zone between humid air coming from the ocean and dry air coming from the Sahara. The demarcation line over land is called the intertropical discontinuity (ITD), shown in Fig. 1d. Above it, another shallow meridional circulation develops related to the SHL. Its lower southerly branch transports moisture across the Sahel, while its upper northerly branch transports dry and warm air, limiting the northward extent of the monsoon^12, 19^. Understanding transport across the monsoon features described below is the main aim of this document.

From the averaged velocity fields, it is possible to roughly identify transport paths of air masses. Nevertheless, the results differ if Eulerian and Lagrangian perspectives are taken into account. For instance, low level monsoon winds into the Guinean coast are expected to bring humidity and moisture into the continent from the Atlantic Ocean, which is a necessary condition for rain to occur inland. The dry air from the Sahara can potentially transport dust, which in turn can act as a catalyst for water vapour condensation and cloud formation^20^, thereby also contributing to rain formation. Many authors have used different methodologies, mostly based on Eulerian analysis, to identify the sources of moisture over West Africa. Most of these studies investigated the sources of water vapour fluxes into West Africa using different sets of data^4,21–24^. The Eulerian approaches are used to estimate the ratio of advected to recycled moisture and to calculate moisture transport from predetermined source regions, although they are unable to identify the moisture source regions directly. The analysis of transport based on Eulerian features, like those displayed in Fig. 1, do not involve real trajectories of atmospheric particles, therefore deep insights into transport processes that occur in the prototypical WAM are lacking. Typically, approaches based on trajectories (Lagrangian approaches) that study air masses and moisture transport are performed on daily based flows^25–34^, in which many of the characteristic Eulerian WAM features just described are absent, since they are only visible in the climatological approach. This is the case for instance of the ITCZ pattern, although other features such as the TEJ are persistent in the daily data. In this way, by averaging over long periods, climatologists find simple models that highlight the essential flow elements of WAM. Lagrangian methods applied to daily data have been an effective diagnostic tool to identify the sources of air masses over a target region^35^. The tracking of particles and their moisture budgets in daily settings suggests that much of moisture comes from local recycling^36^, although the computation of such moisture budget shows some limitations regarding the trajectory accuracy and the time derivative of the humidity used, since unrealistic fluctuations in humidity could be considered as moisture fluxes leading to systematic errors in the tracked humidity budgets. Trajectory analysis has also been used to examine meteorological phenomena like tropical moisture export^37–39^. When applied to West Africa, this type of Lagrangian approaches have shown that a large proportion of tropospheric air masses located over India between 500 and 300 hPa end up over West Africa, after following a direct path through the TEJ^40^.

**Figure 2:**
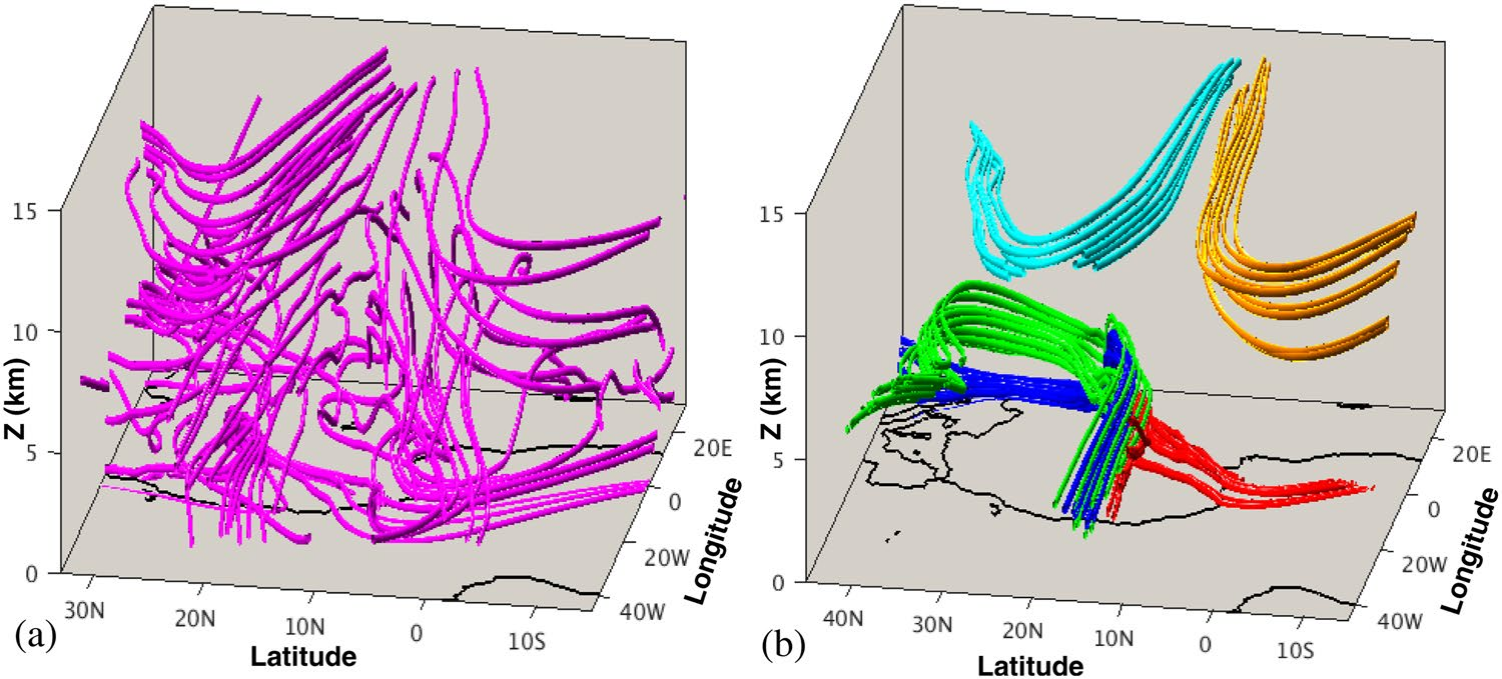
Particle trajectories of the WAM system. (**a**) A set of 54 particle trajectories that evolve forwards and backwards in time for a period of $\tau =15$ days from a regular grid in the plane with constant longitude 10° W; (**b**) five clusters of 10 particles each, that evolve forwards and backwards in time for a period of $\tau =15$ days from regions identified with Lagrangian Coherent Structures on the plane with constant longitude 10° W. Maps in this figure are done with MATLAB R2018b.

In this context, the major goal of this paper is to obtain a comprehensive characterisation of Lagrangian transport across prototypical WAM features, visible only on averaged velocity fields, with the purpose of identifying the role of each WAM element on global transport, with regard not only to moisture transport, but also to dust or aerosols and to discuss how they may influence rain formation^41^.

The study of Lagrangian transport in 3D flows is a complicated task because even in well controlled flows, such as those in lab experiments, it has been demonstrated that fluid parcels follow very intricate paths^42^. The WAM system described in Fig. 1b–d is a 3D flow that also gives rise to genuine entangled flow paths such as those displayed in Fig. 2a. For the study of transport in geophysical flows, the mathematical theory of dynamical systems has played an important role. The pioneering contribution by Aref^43^ on chaotic advection sparked interest in this perspective, inspired by Poincaré’s work. This perspective is based on geometrical structures that separate regions corresponding to trajectories having qualitatively different dynamical fates. In the fluid mechanics community, these geometrical structures have been referred to as Lagrangian Coherent Structures (LCS)^44,45^, and act as material barriers that fluid particles cannot cross. Figure 2b illustrates these ideas. For the same WAM flow that displays the complex structure in panel (a), LCS help to identify regions, in which clusters of particles behave similarly, helping to extract order out of the apparent disorder of panel (a). The fact that there exist regions in this panel where green, blue and red parcels are mixed up, indicate that the separating boundaries between them, adopt intricate shapes.

Many studies of LCS in geophysical contexts have been performed in two-dimensional (2D) settings. For instance, stratospheric flows on the timescale of 10 days are to an initial approximation, adiabatic and frictionless, and thus fluid particles and their trajectories are constrained to remain on surfaces of constant specific potential temperature (isentropic surfaces)^46–48^. A study on the 2D–3D particle motion transition which is observed when passing from the stratosphere to the troposphere is discussed in^49^.

The study of transport processes in 3D flows brings into the discussion issues about the 3D visualisation of Lagrangian structures (see e.g.^50–53^). The methodology used in this study focuses on a Lagrangian method based on the Lagrangian descriptor (LD) known as the *M* function^54–56^. This function has been used to visualise the three-dimensional Lagrangian structures in idealised 3D flows^51,56,57^ and also in atmospheric flows, in the stratospheric polar vortex above Antarctica^49,52,53^. More recently in the context of the Transition State Theory in Chemistry, LDs have been successfully used to picture phase space structures in high dimensional dynamical systems^58–60^.

In this context, this paper exploits the Lagrangian technique based on the function *M*, to describe transport across the summer dynamical features of the West African monsoon. The methodology achieves a partition of the troposphere into regions, containing particles with different origins or fates. This analysis enables the re-examination of all the monsoon elements and their interconnections from a transport perspective by describing how air masses are mutually exchanged and how these exchanges may be linked to rain formation. The paper is organised as follows. First, in section “Data and methodology”, we present the data and the methodology. Afterwards in section “Results”, we discuss the results. Finally, section “Discussion and conclusions” presents the conclusions.

## Data and methodology

### Data

In this study, the ERA-Interim meteorological analysis data from the European Centre for Medium-Range Weather Forecasts (ECMWF)^61^ available at http://www.ecmwf.int is used. In particular, we extract from this source zonal (*u*) and meridional (*v*) wind velocity components, vertical velocity in pressure coordinates ($\omega =dP/dt$), temperature, specific humidity, potential vorticity, geopotential height and surface pressure. These physical variables are available every six hours (00:00, 06:00, 12:00, 18:00 UTC) and are obtained for the month of August in the 1979 to 2015 period^62^. Our focus is on the month of August, because this is the peak of the Monsoon season. The data is daily averaged over this period.

The horizontal data resolution is 0.75° × 0.75° and 60 hybrid-sigma levels along the vertical coordinate. The vertical velocity in pressure coordinates, $\omega$, is transformed into a vertical velocity *w* in m/s, following the procedure described in^49^. Once the 3-D velocities (*u*, *v*, *w*) are obtained for sigma levels, they are interpolated to 41 height levels ranging from 0 to 20,000 m at 500 m intervals. The Supplementary Information provides the three components of the velocities used in this study, in three NetCDF files.

### Methodology

We consider atmospheric particle trajectories in three dimensions, ${\mathbf {x}}(t)$, which evolve according to the dynamical system:

1 $$\begin{aligned} \frac{d{\mathbf {x}}}{dt}={\mathbf {v}}(t,{\mathbf {x}}), \end{aligned}$$

where ${\mathbf {v}}(t,{\mathbf {x}})$ is the velocity field related to the atmospheric winds displayed in Fig. 1. The specific relationship between this velocity and the wind components is found for instance in Eq. (6) in^49^. The use of the velocities displayed in Figure 1, which are obtained from time averages, transforms the study of transport described by Eq. (1), into the study of transport in a stationary system, representative of the monsoon circulation, in which ${\mathbf {v}}(t,{\mathbf {x}})={\mathbf {v}}({\mathbf {x}})$. The study of this system allows a detailed approach to the study of transport induced by the climatological fields described in the “Introduction” section.

To understand transport processes occurring in Eq. (1) from a geometrical point of view, LCS are visualized with the *M* function. This function is built from forward and backward trajectories computed from Eq. (1). Mathematically it is given by the expression:

2 $$\begin{aligned} M({\mathbf {x}}_{0},t_0,\tau ) = \int _{t_0-\tau }^{t_0+\tau }\Vert {\mathbf {v}}({\mathbf {x}}(t;{\mathbf {x}}_0),t)\Vert \; dt \;, \end{aligned}$$

where $\Vert \cdot \Vert$ denotes the Euclidean norm, and $t_0$ and ${\mathbf {x}}_0$ are respectively the initial time and position of the fluid parcel, which is integrated forwards and backward in time for a period $\tau$. The *M* function is obtained by approximating the integral in Eq. (2) by the sum of the lengths (in the Euclidean space) of the segments linking the position of the integrated particle trajectory at two successive time steps. The numerical implementation of Eqs. (1) and (2) in this article exactly follows the procedure described by Curbelo et al.^49^. In this work, since ${\mathbf {v}}$ is a stationary field, the function *M* is also time-independent, which simplifies the analysis of the 3D geometrical structures obtained from it.

**Figure 3:**
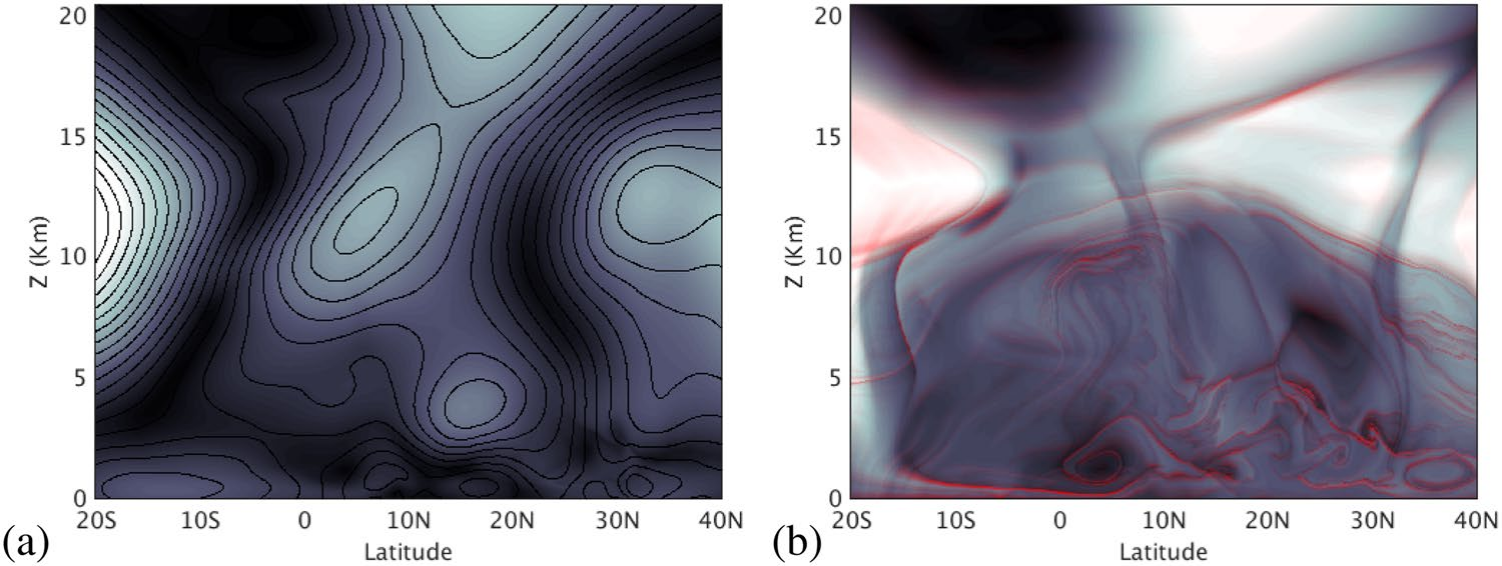
Evaluation of the *M* function at the latitude-altitude plane placed at 10° W using different integration periods. (**a**) $\tau =2$ days. Contours of the zonal velocity are overlapped; (**b**) $\tau =15$ days. The overlapped red tone highlights the singular features that emerge at higher $\tau$ values.

The dependence of the function *M* on $\tau$ deserves further discussion. Figure 3 illustrates this dependence by means of a representation of *M* in the latitude-altitude plane used in Fig. 1c at 10° W. For a small $\tau$ value of 2 days, Fig. 3a shows that the appearance of *M* is smooth and closely follows the contours of the zonal velocity, which are overlapped. Figure 3b displays *M* for a longer $\tau$ period of 15 days. The emergence of singular features is visible. These sharp changes, highlighted in the Figure with the red tone, mark boundaries between regions in which particles have different qualitatively behaviours, and are related to material barriers that particles do not cross. A heuristic argument on why material barriers should be traced out by singular features of *M* is found in^55^. The function *M* measures the lengths of curves traced by trajectories, so it is expected to change abruptly at the boundaries of regions comprising trajectories with qualitatively different evolutions, since this is exactly what the barriers separate. Mancho et al.^56^ provide further details about this. Patterns in Fig. 3b are used to select the clusters of particles displayed in Fig. 2b. They are the LCS that help to extract order structures by identifying regions with an homogeneous transport behaviour. In practice, the integration period necessary to display the required LCS depends on the characteristics of each velocity field. The function *M* reflects the transport history of fluid parcels, and in highly chaotic systems, this history is expected to be increasingly complex for longer $\tau$ intervals, which in turn will be reflected in a more complex structure of the function *M*. We have verified that $\tau = 15$ days is a sufficient choice for our data, and from the physical point of view this is consistent with the time of 30 days used by atmospheric fluid parcels to travel during the month of monsoon peak across the region. Further discussions on the effect of the choice of $\tau$ on the structure of *M* may be found in^55,56^.

The choice of this Lagrangian tool versus others is justified because, despite its simple physical interpretation, its potential for highlighting diverse types of dynamical structures has been rigorously proven in selected examples^57,63,64^. In particular, the “singular features” just discussed are related to dynamical structures linked to highly contracting or expanding regions (connected to mathematical objects called hyperbolic trajectories), while regions with smooth patterns are linked to non-dispersive regions (connected to mathematical objects called tori) that hold matter together. This two-fold capacity has been exploited in the stratospheric polar vortex context in^52,53^. In contrast, other approaches based on tools such as Finite Time Lyapunov Exponents (FTLE), also used in 3D stratospheric studies^65^, highlight only structures related to highly contracting or expanding regions.

The *M* function contains fully 3D Lagrangian structures, and Fig. 3b just captures the intersection of these structures with the vertical slice. Alternatively, in the fluid mechanics context, considerable efforts have been made in representations of 3D LCS, to fully compute them as 2D surfaces embedded in 3D spaces (see for instance^65,66^). This way of representing LCS, which is technically complicated, could also have been adopted in our approach. However, this has not been considered because it would have provided not new transport information to what has already been obtained from our simplest perspective, which consists solely of visualising slices of *M* with different orientations in the geographical domain. On the other hand, our viewpoint has been conjointly adopted in chemical contexts to visualise dynamical structures in high dimensional systems^58–60^, where it also has been proven to be effective.

## Results

**Figure 4:**
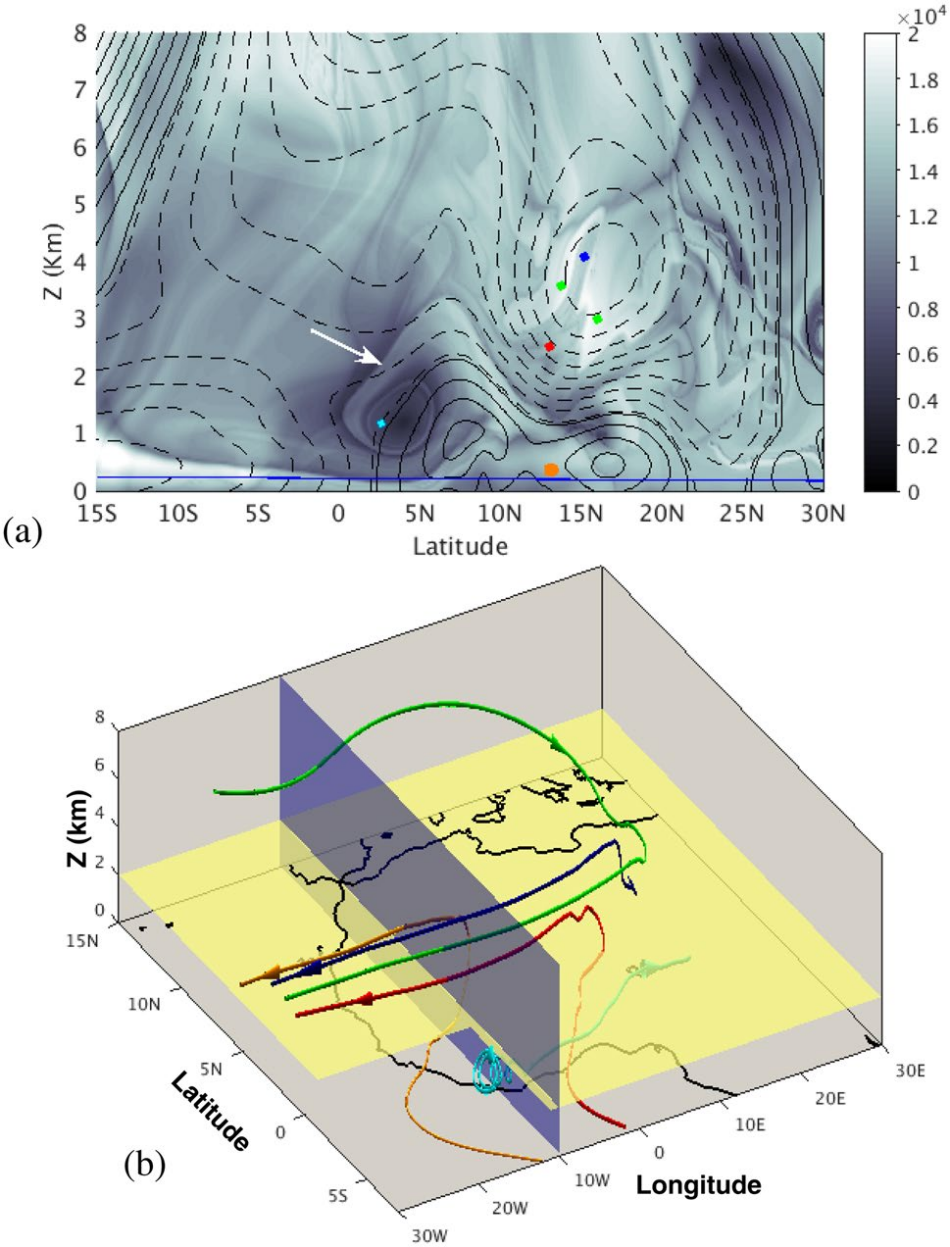
(**a**) A partition of the AEJ by means of the function *M* (LD) which indicates different particle origins. Contour lines of the zonal velocity are overlapped; (**b**) backwards and forwards time evolution of the fluid parcels placed in different domains of the AEJ (red, blue, green) and Gulf of Guinea convective cell (cyan) and the Monsoon flow (orange). Time arrows marking the time direction are placed over the trajectories. A blue line marks the horizontal plane at 0.2 km. Maps in this figure are done with MATLAB R2018b.

Equipped with the tool described in the previous section, we build a geometrical pattern/template on the target region which allows us to perform a comprehensive analysis of air masses sources and fates that affect the major precipitation over West Africa during the peak of the Monsoon season. Figure 4 illustrates this point in detail for the AEJ. Figure 4a overlaps the contours of the zonal velocities on the function *M*, which has been computed with $\tau =15$ days along the vertical and latitude coordinates for longitude 10° W in the same plane as Fig. 1. The broken pattern of *M* over the closed curves of the AEJ indicates the presence in the jet of fluid parcels with a qualitatively different origin or fate: that is, broken contours separate regions with different evolution, either in the past or in the future. In particular, for this case fluid parcels have different origins but are mixed in the AEJ layer. The convoluted forms within it indicate the way in which air masses are mixed. Indeed, three regions are recognised here: particles placed inside the dark grey zone, in upper levels (blue) that are transported into the AEJ from lower altitudes at northern African latitudes; those in lower levels (red) coming from the Gulf of Guinea, and those in the lighter grey zones (green) that come from higher altitudes and northern latitudes, have penetrated into the African continent from the Atlantic Ocean, have then bent at mid-African longitudes and are captured into the AEJ. Indeed, Fig. 4b confirms this point by showing the time evolution of these fluid particles in a longitude-latitude projection. The flight time for these fluid parcels is of 15 days forwards and backwards. For simplicity, only one green particle trajectory is shown, since the other follows a qualitatively similar path and does not add any information. These individual trajectories, displayed in Fig. 4b, are representative of the behaviour of clusters of fluid parcels. This is confirmed by Fig. 2b, in which the blue, red and green clusters are in fact representing fluid parcels in these three regions, with the same colour code.

**Figure 5:**
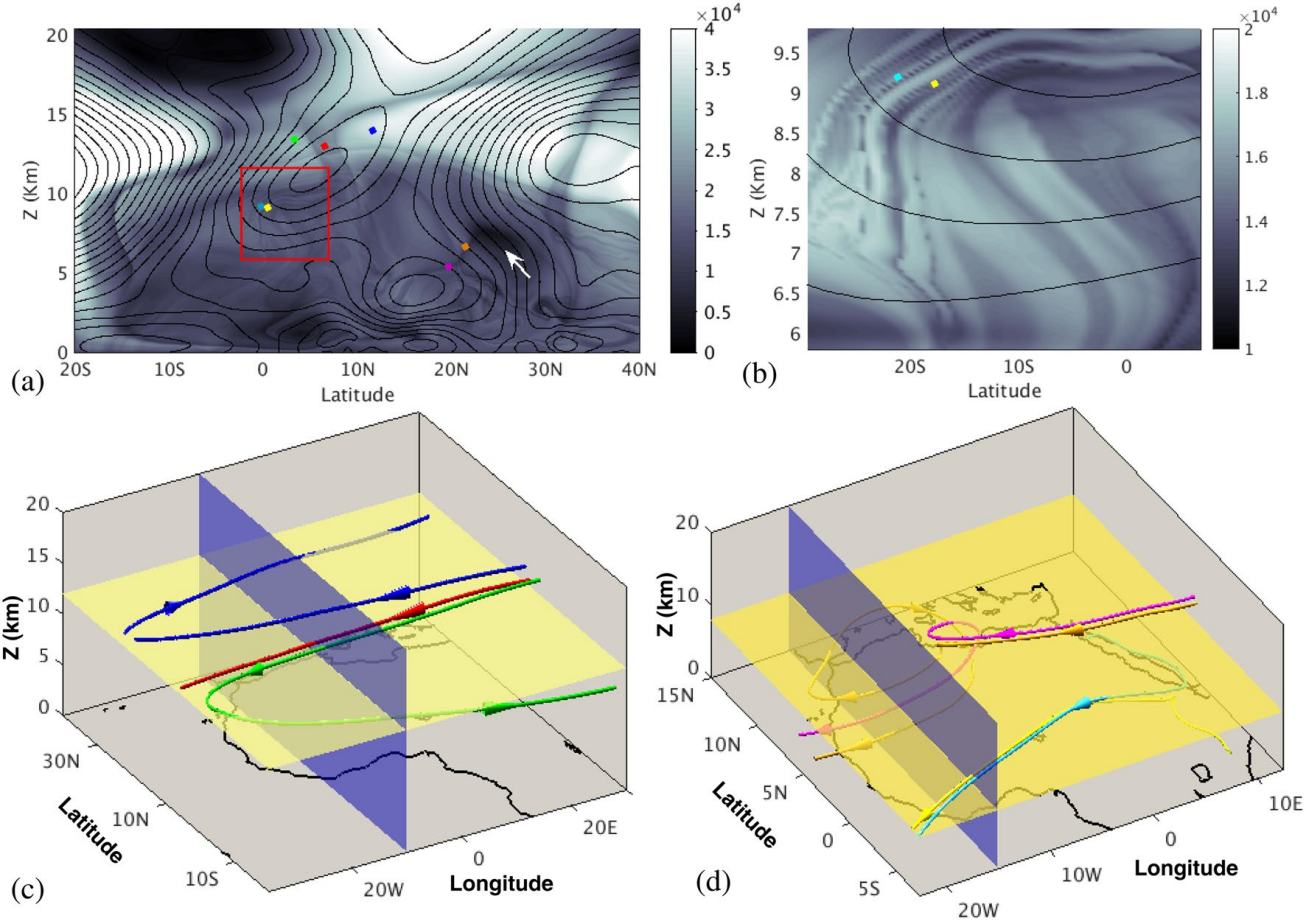
(**a**) A view of the representation of function *M* in the latitude-altitude plane at longitude 10° W. Contour lines of the zonal velocity are overlapped. The TEJ is recognized at latitude $\sim$8° N and altitude $\sim$ 10 km. The red rectagle highlights the region zoomed in panel (**b**); (**b**) a zoom highlighting the lower part of TEJ where the function *M* has a particularly rich structure; (**c**) backwards and forwards time evolution of the fluid parcels placed in different sectors of the TEJ; (**d**) backwards and forwards time evolution of the fluid parcels placed in folds displayed in panel (**b**) and in a selected sharp region of panel (**a**). Maps in this figure are done with MATLAB R2018b.

Rainfall variability is related to the intensity and position of the AEJ^67–69^. The findings shown in Fig.  4a and b indicate that moisture can be brought into the AEJ from the Gulf of Guinea by red-like trajectories. Of course, humidity transportation also requires evaporation at the ocean surface, which is where temperature plays also a role. Additionally, dust coming from inland lower levels can be transported by blue-like trajectories into the AEJ. Both ingredients can contribute to cloud formation and our analysis confirms this capacity. The area of the partitions achieved by the different grey tones, visible in the function *M* in the AEJ, provides a measure of the mixing proportion of these components in this WAM feature. This proportion also has direct connections to cloud formation. Finally, the forward evolution of the trajectories confirms the transport of these elements into the Atlantic, with the subsequent impact on cloud formation on that region and potentially on hurricane formation. Indeed, African easterly waves (AEW) propagating through the AEJ are noted for being precursors to tropical cyclones in the tropical Atlantic^70^. Trajectory analysis and dynamical systems tools in this setting have provided a useful framework in which to describe transport of humid and dry air masses^71, 72^, which are known to play a role in the tropical cyclogenesis formation.

The very dark feature observed in Fig. 4a between approximately latitudes 2.5° N and 7.5° N, marks the southerly Shallow Meridional Cell located over the Gulf of Guinea. The time evolution of the cyan particle allocated in its inner part, displayed in Fig. 4b, confirms that this cell traps particles and that they eventually end up in the interior of the continent. The white arrow in Fig. 4a marks the position of a singular feature highlighting a dynamical boundary, which by warping this region in the Gulf of Guinea area prevents mixing (no transport occurs) with the AEJ. Figure 4b also shows an orange trajectory, which corresponds to a parcel placed in the Monsoon flow. This trajectory penetrates into the continent from the ocean, and then ascends before also ending into the AEJ.

Figure 5 provides a similar analysis to the one described above, but for the TEJ case. Panel (a) displays the partition of TEJ in a grey scale, according to function *M*, overlapped with contours of the zonal velocities. Two regions are identified in dark and light grey tones. Red particles, placed in the dark area are particles trajectories that become straight, while green particles, located in the light area, are particles trajectories that become straight but bend south over the Atlantic towards the Gulf of Guinea, while the blue particle bends towards northern African latitudes. Panel (c) shows a projection in the longitude-latitude-altitude space, which confirms this point. This is consistent with the results discussed by^40^ in which particles trajectories in the TEJ become straight, although our analysis distinguishes regions in the TEJ with different particle fates. Indeed, each particle is just a representative for all the particles placed within each domain. This is confirmed by Fig. 2b in which cyan and orange clusters are linked to the blue and green trajectories of Fig. 5c, respectively.

**Figure 6:**
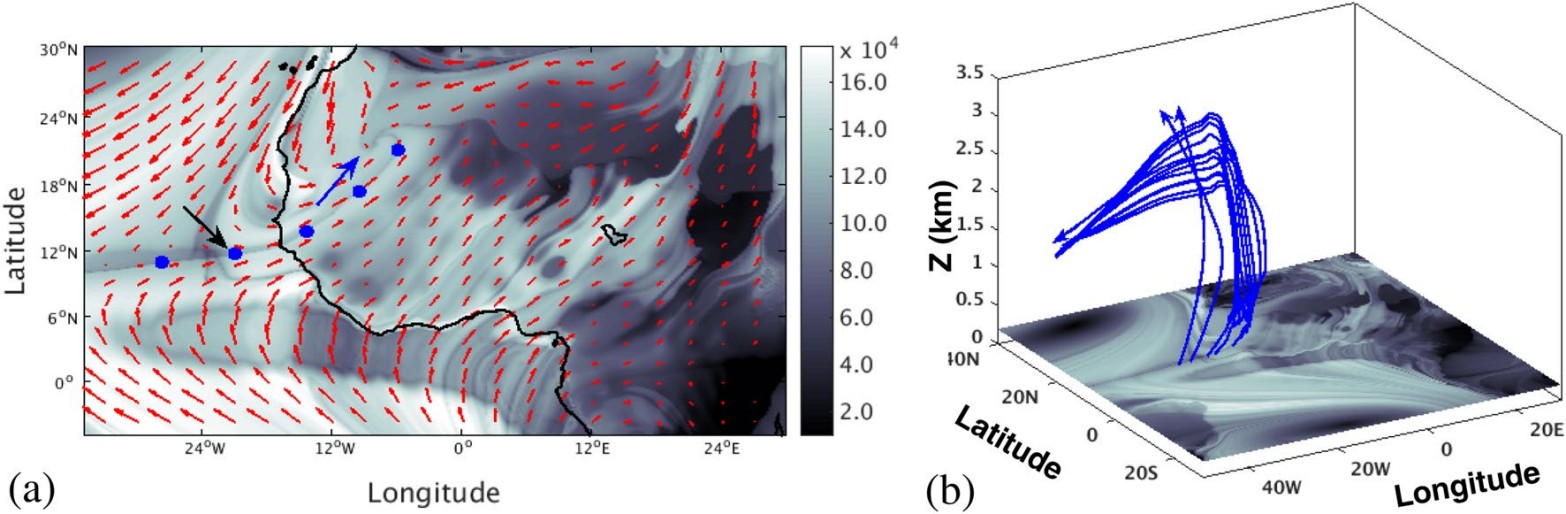
Evaluation of the function *M* for $\tau =15$ at 200 metres height above the sea level. (**a**) The horizontal components of the currents are overlapped in this image; (**b**) the forward time evolution of particles placed along these structures. Time arrows marking the time direction are placed over the trajectories. Maps in this figure are done with MATLAB R2018b.

The red square in Fig. 5a highlights the lower part of the TEJ, where the function *M* has a particularly rich structure and is zoomed in panel (b). This stratified structure is linked to the foldings of an invariant manifold and indicates the presence of a strongly mixing region, where air masses have different sources. Indeed, the two very close particles (yellow and cyan) located there have opposite origins in the lower part of the troposphere, as confirmed by the longitude–latitude–altitude projection in Fig. 5d, one from southern Africa and the other from the north. Although it is not displayed, it occurs analogously with many other air parcels located in that area; there is no any uniformity in their origins, which are very diverse.

Finally, the magenta and orange particles displayed in Fig. 5a are located over a sharp feature in the gray scale, which highlight a boundary for the AEJ. Indeed, the magenta and orange particles are located into a transport pathway from the TEJ to the AEJ. The time evolution of these trajectories displayed in Fig. 5d, confirms that these particles travelled there from the TEJ after circling around the dark feature highlighted with a white arrow in Fig. 5a, and that they evolve into the AEJ.

Figure 6 provides a detailed description of the ITCZ as visible from the computation of the function *M* on a longitude-latitude plane at 200 m height with $\tau =15$ days. The black arrow in the image points to a line where the ITCZ is placed. The blue particles found on that line rapidly ascend (see Fig. 6b). The function *M* also displays a triple convergent point highlighted with a blue arrow. The position of this point is within the hottest point of the SHL displayed in Fig. 1a. The emergence of this type of spiral pattern has been reported to appear in convective fluids heated from below with peaked temperature distributions^73^. The convergent lines separate domains where particles have different origins (not shown), although all particles over these lines end up trapped in the lower parts of the AEJ as illustrated in Fig. 6b. Furthermore, particles located in the flat grey regions surrounding the triple point ascend in a similar way. Consistently with what we have previously described, changes in the grey intensity in this plane denote different behaviours of the particles, and thus the ascending evolution is applicable only to regions sharing the grey colour present in the neighbourhood of the triple point. This image also shows a very pronounced tongue-like structure just over the Gulf of Guinea, which geographically is placed at the position of the southerly Shallow Meridional Cell labelled as SMC in Fig. 1d. This tongue-like feature is the intersection with the horizontal plane of the sharp line that appears at low altitudes in Fig. 4a. For reference a horizontal blue line at 200 m is highlighted in Fig. 4a and shows two intersection points with this sharp feature. The tongue is therefore placed below the convective cell that has been associated to the cyan fluid parcel in Fig. 4. The southern boundary of this structure is located approximately above the Equator, to the North of the cold tongue observed in the sea surface temperature climatological series, displayed in Fig. 1a. The higher pressure associated with the local cold temperature, would imply northwards fluid parcel motions close to the sea level and this is confirmed by the circulation direction of the cyan trajectory in Fig. 4b. Our interpretation is therefore that this dynamical structure could be closely linked to the sea surface temperature pattern.

**Figure 7:**
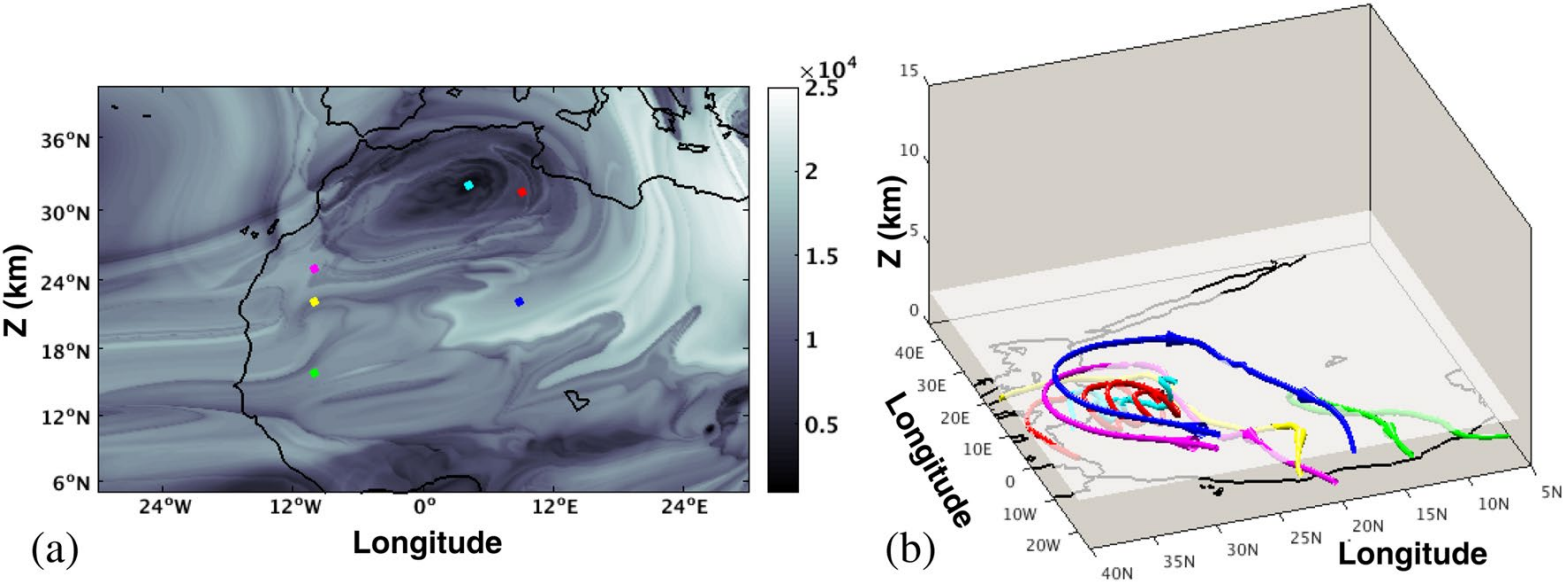
Evaluation of the function *M* for $\tau =15$ days at 2 km height above the sea level. Red, blue, cyan, magenta, yellow and green represent particles located at different domains of the partition induced by *M*. Time arrows marking the time direction are located over the trajectories. Maps in this figure are done with MATLAB R2018b.

The computation of the function *M* on the longitude-latitude plane at 2 km height with $\tau =15$ days is displayed in Fig. 7. The grey area where magenta and yellow particles are located is a dynamical structure found above the surface SHL. This area displays a rich *M* structure, which reflects different fluid parcels origins. The evolution in this region is somewhat more complex than what is described at 200 m, because at this level not all fluid parcels are ascending from the bottom. However, there are also fluid parcels coming from above, describing paths such as the magenta trajectory in Fig. 7, while others, such as the yellow one, are coming from lower heights. To the east of this region, there is a light grey structure where particles like the blue one evolve according to the arrows depicted over the trajectories in Fig. 7b, and have arrived at that point in a motion that was descending some days earlier. All three particles, magenta, yellow and blue, share the same destination, since they all end in the AEJ. It is interesting to note that all three describe a trajectory that wraps clockwise the very dark feature located at the northeast of the surface SHL, where the cyan and red particles are found. Such clockwise circulation indicates that the very dark feature is the mid-level signature of the anticyclone surmounting the SHL^74^, which is associated with the diverging flow at the top of SHL and contributes to the maintenance of the African Easterly Jet (AEJ), modulating its intensity^18^.

Further to the south, there are fluid parcels coming from the bottom (green path) that also end in the AEJ, but which do not circle the anticyclonic dark feature. Particles in red located in the very dark feature, evolve following a clockwise circular path with a general ascending tendency, but also with very pronounced ascending–descending cycles. In the very dark interior part cyan particles also evolve ascending with less pronounced circular motions. For the studied $\tau$ period, the cyan or red particles located here do not approach neither backwards nor forwards in time to the neighbourhood of the surface SHL, and are domains disconnected from the AEJ. This suggest that there is no transport between the SHL at the surface and its surmounting anticyclone.

## Discussion and conclusions

In this paper we revisit the description of transport across classical West African Monsoon features from a Lagrangian Coherent Structures perspective. We find that with the support of this tool, which has not been used before in this context, we are able to identify not only the main characteristics flows but also new pathways and connections in the area.

The mathematical tool that we have used to display the dynamical template describing transport are Lagrangian Descriptors, in particular, the so called function *M*. With the aid of this tool we are able to identify boundaries that separate zones where particles have different qualitative behaviours: that is, different origins or fates. This geometrical representation has advantages with respect to the plain representation of trajectories in the troposphere. Indeed, with the latter approach it is extremely difficult to find structures of order because trajectories follow very complex paths that intermingle with each other and information about transport is difficult to extract. On the other hand, our analysis provides a guide that allows a precise selection of initial conditions for comparison purposes, which has enabled us to find new transport routes, as well as providing an integrated vision of transport. Our discussion considers a forward and backward travel time for fluid parcels of $\tau =15$ days consistent with the transport occurring during the peak month season. During this travel time a rich Lagrangian geometrical structure (LCS) emerges and transport related to some of its features has been discussed.

The integrated transport vision provided by this method has allowed us to identify mixing patterns in the AEJ region that are linked to pathways to sources of humidity and dust. Cloud formation coming from dust sources in West Africa has been discussed by Wiacek et al.^75^, who found that the mean region of cloud formation is consistent with the AEJ location. There is a strong relationship between the AEJ and Saharan mineral dust (SMD) and AEJ and West African precipitation (WAP). A recent paper^76^ has found how the combined effect of WAP and SMD determines the changes in the AEJ. Thus, it is very important not only to characterise the AEJ but also the sources of dust and rain.

Our approach has enabled us to progress from these findings by sketching an argument based on the LCS patterns about how to quantify the proportion of these elements of dust and humidity on the AEJ. Since dust is a candidate that may act as a catalyst for water vapor condensation, this finding could lead to important consequences in cloud formation. Using this Lagrangian approach, the clockwise circulation associated with the diverging flow at the top of the SHL is clear, and has been found to maintain the African Easterly Jet (AEJ)^18^. Transport pathways detected from LCS unveil connections between the TEJ and the AEJ. Indeed, after following a circulation pattern in the troposphere above the SHL, trajectories found on the TEJ end up in the AEJ. Note that this connection shows particles subsiding from the TEJ to the AEJ, contrary to what could be inferred from the Eulerian perspective^14^. Unlike suggestions by other authors^4^, we have not detected a Shallow Meridional Cell associated with the Saharan heat low, and the connection is found through the AEJ. These connections open up new possibilities for the influence and impact of the TEJ on African rainfall, which is a topic currently under discussion^77,78^. Additionally, through the analysis of a folding structure visible in the *M* function at the lower part of the TEJ, connections between the Earth’s surface and the TEJ have been found. It should be pointed out that these particles leave the surface over eastern Africa, rather than over the Gulf of Guinea, as could be inferred from the Eulerian perspective^14^.

Finally, our methodological approach very clearly highlights other well-known pathways in the WAM system. Our results are very promising, since we foresee a great impact on the assessment of drought periods in this region, as well as in other monsoon regions if they are studied from this perspective.

## Supplementary information

Supplementary Legend.

Supplementary Information 1.

Supplementary Information 2.

Supplementary Information 3.
