# Supplementary material for: Transport pathways across the West African Monsoon as revealed by Lagrangian Coherent Structures
Source: Sci Rep. 2020 Jul 27;10:12543. doi: 10.1038/s41598-020-69159-9 (PMC7385109; doi:10.1038/s41598-020-69159-9)
Supplement: Supplementary file 1 — Supplementary Legend. [file 41598_2020_69159_MOESM1_ESM.doc]

**Transport pathways across the West African Monsoon as revealed by Lagrangian Coherent Structures**

Coumba Niang, Ana Maria Mancho,Víctor José García-Garrido, Elsa Mohino, Belén Rodriguez-Fonseca,Jezabel Curbelo

Supplementary material includes the data used for our study in the following files

**1) U_height-1979-2015-monmean_Aug.nc**

This file contains the westward component of the velocity field averaged over the month of August in the period that runs from 1979 to 2015. The averaged data is ERA Interim from the ECMWF.

**2) V_height-1979-2015-monmean_Aug.nc**

This file contains the southward component of the velocity field averaged over the month of August in the period that runs from 1979 to 2015. The averaged data is ERA Interim from the ECMWF.

**3) W_height-1979-2015-monmean_Aug.nc**

This file contains the vertical component of the velocity field averaged over the month of August in the period that runs from 1979 to 2015. The averaged data is ERA Interim from the ECMWF.

All the NetCDF files contain the necessary information for representation and use.
